# Can Microbial Consortium Applications Affect Yield and Quality of Conventionally Managed Processing Tomato?

**DOI:** 10.3390/plants12010014

**Published:** 2022-12-20

**Authors:** Giovanna Marta Fusco, Andrea Burato, Alfonso Pentangelo, Mariateresa Cardarelli, Rosalinda Nicastro, Petronia Carillo, Mario Parisi

**Affiliations:** 1 Department of Environmental, Biological and Pharmaceutical Sciences and Technologies, University of Campania “Luigi Vanvitelli”, Via Vivaldi 43, 81100 Caserta, Italy; 2 CREA Research Centre for Vegetable and Ornamental Crops, Via Cavalleggeri 25, 84098 Pontecagnano Faiano, Italy; 3 Department of Agriculture and Forest Sciences, University of Tuscia, Via San Camillo de Lellis snc, 01100 Viterbo, Italy

**Keywords:** *Solanum lycopersicum*, mycorrhizae, *Trichoderma*, plant growth-promoting bacteria, biostimulants, fruit quality

## Abstract

Three commercial microbial-based biostimulants containing fungi (arbuscular mycorrhizae and *Trichoderma* spp.) and other microrganisms (plant growth-promoting bacteria and yeasts) were applied on a processing tomato crop in a two-year field experiment in southern Italy. The effects of the growing season and the microorganism-based treatments on the yield, technological traits and functional quality of the tomato fruits were assessed. The year of cultivation affected yield (with a lower fruit weight, higher marketable to total yield ratio and higher percentage of total defective fruits in 2020) and technological components (higher dry matter, titratable acidity, total soluble solids content in 2020). During the first year of the trial, the consortia-based treatments enhanced the soluble solids content (+10.02%) compared to the untreated tomato plants. The sucrose and lycopene content were affected both by the microbial treatments and the growing season (greater values found in 2021 with respect to 2020). The year factor also significantly affected the metabolite content, except for tyrosine, essential (EAA) and branched-chain amino acids (BCAAs). Over the two years of the field trial, FID-consortium enhanced the content of proteins (+53.71%), alanine (+16.55%), aspartic acid (+31.13%), γ-aminobutyric acid (GABA) (+76.51%), glutamine (+55.17%), glycine (+28.13%), monoethanolamine (MEA) (+19.57%), total amino acids (TAA) (+33.55), EAA (+32.56%) and BCAAs (+45.10%) compared to the control. Our findings highlighted the valuable effect of the FID microbial inoculant in boosting several primary metabolites (proteins and amino acids) in the fruits of the processing tomato crop grown under southern Italian environmental conditions, although no effect on the yield and its components was appreciated.

## 1. Introduction

The tomato (*Solanum lycopersicum* L.) ranks third in vegetable crop production after potatoes and cassava, reaching a worldwide yield of around 187 million tons on a cultivated area of almost 5.05 million hectares in 2020 [1,2], and represents an excellent source of nutrients (Ca, K, Mg, organic acids and simple sugars) and health-promoting compounds including vitamins (B, E, C, K), phenolic compounds and carotenoids [3,4,5].

Although lycopene [6], polyphenols and ascorbic acid are known as the most important health-related compounds in tomato fruits [3], also noteworthy is their content of γ-aminobutyric acid (GABA). The biological role of this non-proteinogenic amino acid has not yet been fully clarified; nevertheless, GABA is widely recognized as a bioactive and functional compound in humans, as it mainly acts as an inhibitory neurotransmitter and as a restraint for blood pressure in hypertensive patients [7].

The quality of tomatoes is strongly affected by the genetic background of the variety; however, several external factors affect fruit composition such as agronomic techniques, growing conditions, production methods, harvest time and storage [8,9,10,11].

Among agricultural techniques, the application of microbial consortia containing arbuscular mycorrhizae (AMF), *Trichoderma* fungi and plant growth-promoting bacteria (PGPB) has been successfully used to improve plant growth, yield, abiotic stress tolerance and the nutritional and functional quality of tomato fruit [12,13,14,15,16,17,18]. Their applications are of particular interest in the context of climate change (referring to shifts in air temperature patterns, timing and amount of rainfall and soil salinization) [19,20,21] to maintain or increase tomato yield and boost fruit quality attributes [12,22,23,24].

Arbuscular mycorrhizae establish a mutualistic symbiosis by creating specialized and highly branched hyphae inside the cell lumen of the roots, called arbuscules, which are efficiently used for nutrient exchange (in particular, P and N) [25]. The relationship is beneficial for both partners, as AMF enhance mineral nutrient exchange between the soil and the host, while the plant supplies carbohydrates and represents a safe and necessary home for the growth and reproduction of the guest [25].

*Trichoderma* establishes mutualistic endophytic relationships with several plant species enhancing plant growth, yield and the alleviation of abiotic stress [26]. *Trichoderma* is widely applied for its biocontrol activity against the main root pathogens, which is exerted through mycoparasitism, antibiosis and competition for space and nutrients [26].

Plant growth-promoting bacteria naturally populate the rhizosphere and colonize the root tissues of the host plant [27]. They produce and release several compounds, such as phytohormones and volatiles, to facilitate nutrient uptake by the plant, lower the ethylene level in the plant and stimulate induced systemic resistance (ISR). Moreover, they exert a biocontrol action against diseases through the stimulation of beneficial symbioses or the production of antibiotic compounds [28]. Considering the growing attention on biostimulants [29,30,31], several microbial consortia (with different fungi or/and bacterial composition and formulation) are continuously released on the market as beneficial products for tomato crops.

These commercial microbial inoculants, mainly grouping AMF, fungi and free-living bacteria that act as biofertilizers [32], may result in more robust and efficient fruits, with a longer shelf life and sustainability and higher rate of metabolism and growth, thus incurring a lower cost of mass multiplication. They provide a simplified system encapsulating multiple known beneficial properties at one time, difficult to untangle and recognize in one single species [33]. Indeed, the synergism among microbes is the most essential feature responsible for the higher rate of success in field [34]; in fact, their cross talk allows them to work as a community with high performance in symbiosis. Besides, the best formulation of microbial consortia can match and synergize the beneficial effects of multiple microorganisms on plants’ resource use efficiency, growth and protection against multitargeted pathogens thus increasing the yield and/or quality of the plant products [35].The aims of this work were to (*i*) assess the effect of three commercial products on the yield components, technological traits and functional quality of processing tomato fruits, and (*ii*) identify the biostimulant better enhancing tomato fruit quality on a conventionally managed processing tomato crop in the most important area of production in southern Italy (Apulia region).

## 2. Results

### 2.1. Yield and Its Components

As reported in Table 1, green yield (GY) was higher in 2021 (25.31 t ha^−1^) with respect to 2020 (13.55 t ha^−1^). Conversely, rotten yield (RY) was lower in 2021 (3.73 t ha^−1^) compared to the previous year (6.13 t ha^−1^). No significant variations were found for marketable yield (MY) (80.89 t ha^−1^ in 2020 and 79.52 t ha^−1^ in 2021) and total yield (TY) (100.58 t ha^−1^ in 2020 and 108.56 t ha^−1^ in 2021), while the MY to TY ratio was significantly higher in 2020 than 2021 (80.89% and 73.74%, respectively). Mean fruit weight (FW) (69.91 g, as mean) was greater in 2021 than in 2020 (71.63 g vs. 68.19 g), although no effect of this yield component was appreciable on MY across the two years of the field experiment.

Regarding fruit defects, microbial biostimulants did not have significant effects in comparison with the untreated control (CTRL) (4.42% of sunscald fruits (SsF), 14.67% of fruits with viral symptoms (VrF), accounting for 19.08% of total defective fruits (TDF), on average). However, valuable differences between the two years were observed. Indeed, in the first year the percentages of SsF and VrF were higher (5.83% and 26.50%, respectively) than in 2021 (3.00% and 2.83% for SsF and VrF, respectively).

Finally, no significant effect of treatment × year interaction was found for the all yield-related traits reported in Table 1.

### 2.2. Chemical and Technological Traits

Fructose, total sugars (Tsu) (as sum of glucose (Glc), fructose (Fru) and sucrose (Suc)), starch (Sta) and lycopene (Lyc) contents were increased in 2021 with respect to 2020 (+67.32%, +32.13%, +31.25% and +15.81%, for Sta, Lyc, Fru and Tsu, respectively) (Table 2). Conversely, a higher content of sucrose was detected in 2020 compared to 2021 (0.82 µg g^−1^ and 0.23 µg g^−1^, respectively). No significant variation of Glc and polyphenol (PP) contents was found in tomato fruits produced in both years.

Micosat F Tab Plus and FID induced a higher sucrose level with respect to EKO biostimulant (0.73, 0.64 and 0.27 µg g^−1^, respectively), although the MIC, EKO and FID treatments did not significantly differ from the control. The EKO biostimulant also increased Lyc content in comparison with the CTRL (7.99 and 6.56 mg g^−1^, respectively) (Table 2).

Regarding the other relevant antioxidant compounds of tomato fruits, PP were not affected by the different biostimulant treatments in both years of the field trial.

The applications of microbial consortia significantly affected the Suc content compared to CTRL in 2020. In particular, MIC increased Suc by 83.1%, while the EKO inoculant worsened this trait (−63.4%) with respect to CTRL. (Table 2).

With respect to the technological quality of the tomato fruits, the titratable acids (TtA), soluble solids (SSC) and fruit dry matter (DM) contents were negatively affected by the 2021 growing season (0.41 g% of citric acid, 5.21 °Brix and 6.03 g%, respectively) compared to 2020 (0.51 g% of citric acid, 5.41 °Brix and 6.53 g%, respectively), while pH was higher in 2021 (4.56) with respect to 2020 (4.41). No statistical difference was recorded among the three treatments and CTRL for all technological parameters. Conversely, a significant effect of Treatment x Year interaction was found for SSC, which was improved by biostimulants (+8.75%, +9.94%, +11.33% for MIC, EKO and FID, respectively) with respect to untreated CTRL in the first year of the experiment.

### 2.3. Primary Metabolite Content

As for most of the previously analyzed traits, the year of cultivation significantly affected all evaluated metabolite contents, except for tyrosine (Tyr), essential (EAA) and branched-chain amino acids (BCAAs). In fact, the contents of proline (Pro), histidine (included in the EAA), asparagine (Asn), alanine (Ala), serine (Ser), ornithine (Orn) and glycine (Gly) in tomato fruits in 2020 were +108.51%, +86.51%, +72.52%, +69.32%, +51.01%, +50.01% and +21,44% higher than those of 2021. On the contrary, the soluble protein (Prot), γ-aminobutyric acid (GABA), aspartic acid (Asp), monoethanolamine (MEA), glutamine (Gln) and glutamic acid (Glu) contents were lower in the first year of the field trial (−63.6%, −33.1%, −30.9%, −30.3%, 29.9% and 21.9%, respectively).

In 2021, the FID biostimulant increased Prot (8.47 mg g^−1^) and total amino acid (TAA) contents (98.04 µmol g^−1^) in comparison with untreated CTRL (5.05 mg g^−1^ and 57.19 µmol g^−1^, respectively) (Table 3). Moreover, in the same year, the EAA (including isoleucine, leucine, lysine, methionine, phenylalanine, threonine, tryptophane and valine) and BCAA (including isoleucine, leucine and valine) contents were improved by the FID biostimulant, which significantly differed from the EKO and MIC biostimulants and the untreated CTRL (+80.21% and +115.12% compared to CTRL, respectively) (Table 3).

**Figure 1:**
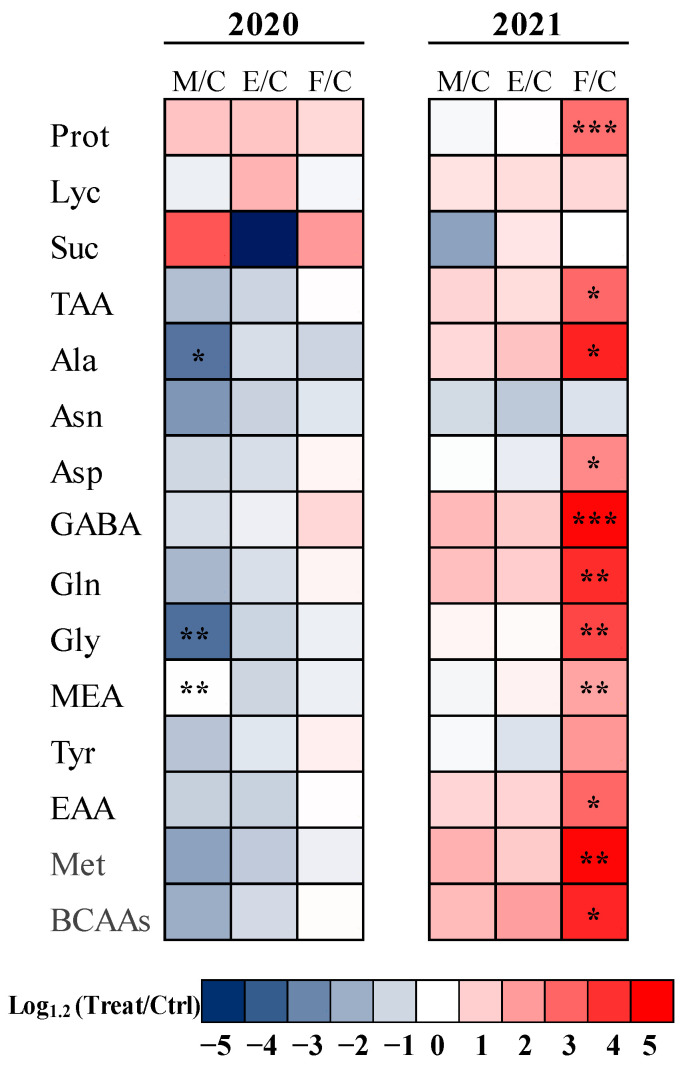
Heat map analysis summarizing statistically significant tomato fruit responses to different commercial biostimulant treatments (M = Micosat F Tab Plus, E = EKOprop NX, F = Fidelius) compared to C = control. Results were calculated as Logarithm base 1.2 (Log1.2) of treatments/control in the two years of agronomic cultivation (2020–2021) and visualized using a false color scale with red indicating an increase and blue a decrease. No differences were visualized by blank squares. *, **, *** = significant at *p* ≤ 0.05, 0.01 and 0.001, respectively, and indicate significant differences between treatment × year according to Tuckey’s range test (*p* ≤ 0.05).

In Figure 1, a heat map analysis summarizing statistically significant tomato fruit responses to different commercial biostimulant treatments (MIC, EKO and FID) compared to the control is represented. Based on this analysis, significant decreases in Ala and Gly content were found for the MIC treatment with respect to the untreated CTRL in 2020 (−46.41% and −46.62%, respectively). A great effect of FID was noticed in 2021; indeed, this microbial consortium boosted the content of GABA, Glu, Gly, EAA (in particular that of arginine), Asp and MEA (+136.71%, +110.12%, +95.62%, +80.11%, +53.52% and 39.24%) in comparison with the respective controls (Figure 1).

## 3. Discussion

Microbial-based biostimulants are often applied by tomato growers to improve yield and boost fruit quality under different environmental conditions. Moreover, these biostimulants have great effects under abiotic stress conditions (reduced water availability, soil salinity and low endowment of nutrients), representing a valuable resource to mitigate the threatening consequences of climate change on processing tomato crops [19,20,29,36,37].

In the present work, the effect of three commercial microbial-based biostimulants containing AMF, *Trichoderma* fungi and PGPB (Micosat F Tab Plus, EKOprop NX and Fidelius) on the yield, technological traits and functional quality of tomato fruits was assessed.

### 3.1. Effect of Different Microbial Consortia on Yield and Technological Quality of Tomato Fruits

Most of the evaluated attributes were significantly affected by the growing season. No variations in total and marketable yield were found between 2020 and 2021, although significant differences in terms of phytopathological problems were observed in the two experimental years. In fact, during the crop cycle of 2020, valuable infections by *Fusarium* spp. (a fungi causing tracheomycotic disease) and *Tomato Spotted Wilt Orthotospovirus* (TSWV) were detected on tomato plants. The TSWV incidence was measured at the harvest as the number of fruits with viral symptoms (VrF), accounting for up to 26.5% in the first year of the field trial. Infections by TSWV and *Fusarium* soil-born fungi accelerated the tomato crop cycle, causing earlier plant ageing and fruit ripening (higher marketable and rotten yield to total yield ratios in 2020). Furthermore, plant decay and leaf fall, as an effect of both diseases, resulted in an over-exposition of the fruits to the sunlight and therefore in higher percentage of sunscald fruits in 2020 with respect to 2021. A higher percentage of green yield to total yield (GY/TY ratio) in 2021 than the previous year was related to an early harvesting as well as the good phytosanitary status of the plants and a great availability of water by rainfall and irrigation supplies. According to our findings, Colla et al., 1999 [38], reported a positive effect of increases in irrigation volumes on fruit coverage and fruit weight, as well as on the reduction of sun burning the fruits. The same relationship between mean fruit weight and water regime was also reported by Di Cesare et al., 2012, and Patanè and Saita, 2015 [39,40]. The variations in fruit dry matter (DM), pH, titratable acidity (TtA) and soluble solids content (SSC) could be related to a different irrigation volume between the years of the experiment. Indeed, different works have highlighted a worsening in DM, SSC and TtA attributes under increasing water supplies in the processing tomato crop [38,39,40,41].

In the first year of the experiment, all the microbial-based biostimulants positively affected SSC in comparison with the untreated control, in full accordance with previous works [42,43]. Soluble solids represent a crucial parameter in the quality evaluation of processing tomato fruits, and thus for their profitability, as the optimization of industrial processes (e.g., the production of tomato paste) is highly dependent on the soluble solids content of the incoming fruits [44].

To summarize, the biostimulant consortia were not effective in improving tomato yield compared to the untreated control. To determine whether a biostimulant application is cost-effective, it is necessary to consider not only the cost of biostimulants in terms of use (purchase and distribution), but also their effect on the yield (volume and value of production). For this reason, no economic evaluation has been taken into account in our work.

### 3.2. Effect of Different Microbial Consortia on Biochemical Properties of Tomato Fruits

The EKO treatment over the two years of the experiment significantly increased the lycopene content, which is the main antioxidant molecule of the tomato fruit, responsible for the bright red color and the quality and the shelf-life of the tomato and tomato-derived products [23]. This carotenoid is able to detoxify reactive oxygen species (ROS), particularly hydroxyl radicals, and stimulate antioxidant enzymes such as superoxide dismutase, glutathione peroxidase and glutathione reductase in plant cells, providing an increased resistance to oxidative stresses (e.g., salinity, drought, high light, etc.) and a shelf-life extension. Moreover, it can exert the same action in animal cells, thus improving the nutritional and nutraceutical properties of tomatoes [45], preventing oxidative stress-mediated carcinogenesis [46,47,48]. In EKO, *Glomus* spp. are present and have already been found to be able to enhance lycopene content, alone in Moneymaker tomato fruits [49], or in combination with *Trichoderma* [50].

In the year 2020, the same biostimulant also resulted in a decreasing sucrose content, and was probably a source of carbon skeletons and/or energy for the synthesis of lycopene, since very high costs for the synthesis of organic compounds for oxidative stress or osmotic protection (50–70 moles ATP for mole) are required [51], whereas sucrose underwent a non-statistically significant but effective accumulation in fruits from plants under the FID and MIC treatments compared to the control ones, thus probably exerting a function as an osmolyte and stabilizer of macromolecules, as previously shown in the leaves and roots of rice plant under water shortage [52]. Two commercial preparations, containing six and eight different AMF species, among them *Funneliformis mosseae* (ex. *Glomus mossae*) and *Rhizophagus intraradices* (ex. *Glomus intraradices*), were also able to increase soluble sugars in the fruits of AMF-inoculated tomato plants of the variety Admiro F1 when cultivated under a high phosphorus content of nutrient solution [53].

The FID biostimulant in 2021 was able to induce a strong increase in γ-aminobutyric acid (GABA) in tomato fruits. This non-protein amino acid is mainly synthetized by the decarboxylation of glutamate catalyzed by glutamate decarboxylase. However, the increase in GABA did not determine a decrease in glutamate that remained stable. The treatment on the hybrid plum tomato Pixel with *T. harzianum* (present in FID) also enhanced the content of GABA in fruits without determining a decrease in its precursor, glutamate [54]. The stability in the content of the latter amino acid is of pivotal importance for tomato flavor and fruit palatability, since it elicits an intense umami taste [54]. A commercial consortium containing *Rhizoglomus irregulare* BEG72 and *Funneliformis mosseae* BEG234 also increased GABA in the “Lucariello” biotype belonging to the “Pomodorino del Piennolo del Vesuvio” local Italian tomato variety [54]. In its zwitterionic form, GABA may act as an osmolyte balancing the water potential during cellular dehydration, and as an antioxidant stabilizing and protecting the structure and function of macromolecules [55]. Moreover, GABA is widely recognized as a bioactive and functional compound in humans, as it, in addition to its role as a hypotensive, can increase immune functions under stress, prevent diabetes and cancer and control blood cholesterol levels [56,57,58]. Over two years, the FID treatment was also able to increase tyrosine content, which was independent from protein degradation, since a higher soluble protein (Prot) content was induced by the same biostimulant in comparison with the control. Tyrosine could function as a precursor in the synthesis of tocopherols and other lipophilic antioxidants [59], but it could itself function as an antioxidant as suggested by Shahidi and Zhong, 2007 [60]. Fidelius only in 2021 increased the content of monoethanolamine (MEA), a metabolite deriving from the decarboxylation of the photorespiratory serine and fundamental for the synthesis or regeneration of membrane phospholipids, functions that allow the plant to have a greater resistance to ROS. *Trichoderma harzianum*, as well as the biopolimer + 6-pentyl-α-pyrone, a *Trichoderma* secondary metabolite, have also been found to determine an increase in MEA in the hybrid plum tomato Pixel [54]. Therefore, the application of FID allowed tomato plants to reuse photorespiratory amino acids and ammonium to synthesize this useful metabolite and decrease the pressure of photorespiration on stressed plants [54].

An increase in the content of total amino acids (TAA), alanine (Ala), glutamine (Gln) and essential amino acids (EAA), in particular methionine (Met) and branched-chain amino acids (BCAAs leucine, isoleucine and valine together) was detected under FID treatment over the two years, particularly in 2021. Similar results were obtained by using AMF *Glomus* spp. consortia on cherry tomato fruits [23]. Moreover, *G. mossae* was able to increase free the amino acid content (i.e., glutamine and asparagine) in the Micro Tom tomato by upregulating the transcription of genes involved in N and C metabolism [61]. Alanine is a precursor of CoA and is also implicated in membrane phospholipid synthesis, fatty acid synthesis and degradation, and plays an active role in the secondary metabolism and plant response to biotic and abiotic stresses [62]. The increase in glutamine under abiotic stress has been reported in wheat [63,64], as well as its possible role in osmotic adjustment and macromolecule protection [65,66]. Glutamate content, which can affect tomato flavor and fruit palatability, was not affected by the biostimulant treatments [54]. The EAA content was also enhanced by the FID treatment, principally in the second year of the experiment. Their improvement, and in particular that of the BCAAs, could be useful both as a compatible compound and as an alternative electron donor for the mitochondrial electron transport chain [67,68,69]. Moreover, BCAAs have a high nutraceutical value since recent findings have demonstrated that they are able to decrease oxidative stress in mice and rats by an unknown mechanism [70,71]. The accumulation of methionine can be correlated to its role as a precursor of BCAAS, in particular isoleucine [68].

## 4. Materials and Methods

### 4.1. Location

An agronomic trial was carried out in an open field at Foggia (41°32′45.4″ N; 15°36′18.4″ E) (Southern Italy) during a two-year period (2020–2021) in a typical Haploxerepts soil (Soil Taxonomy; USDA, 2014) [72]. Physical and chemical soil properties were as follows: sand 41%, silt 20%, clay 39%, limestone 19.3 g kg^−1^, pH 7.52, organic matter 22.9 g kg^−1^, total nitrogen 1.66‰, P_2_O_5_ 10.6 mg kg^−1^, K_2_O 268 mg kg^−1^, C/N ratio 7.99 and Cation Exchange Capacity (CEC) 25.1 meq 100 g^−1^.

### 4.2. Meteorological Data

The climate of this region was typically Mediterranean. The mean maximum and minimum air temperatures and total rainfall during the cropping cycles (April–May to August–September) were 29.5 °C and 17.0 °C and 89 mm for the year 2020, and 31.3 °C and 17.5 °C and 120.2 mm for the year 2021, respectively (Supplementary Materials Figure S1).

### 4.3. Experimental Design and Microbial-Based Treatments

The processing tomato hybrid “Heinz 1534” (Furia Seed, Monticelli Terme (PR), Italy), showing determinate habitus and blocky/round fruits, was adopted in this experiment. Seedlings were transplanted on 19 May 2020 and 29 April 2021 in paired rows, with a 0.38 m spacing along the rows and 0.40 m among the rows. The distance between pairs was 1.40 m, with a plant density of 2.24 plants m^−2^.

The N, K and P requirements were calculated on the basis of soil analysis and the expected fruit yield (under these environmental conditions), and were supplied before transplanting and during the crop cycle according to the phenological growth stages (125 kg ha^−1^ of N, 76 kg ha^−1^ of K_2_O and 109 kg ha^−1^ of P_2_O_5_ in 2020; 80 kg ha^−1^ of N, 140 kg ha^−1^ of K_2_O and 107 kg ha^−1^ of P_2_O_5_ in 2021).

Irrigation scheduling was based on evapotranspiration of the crop (ETc) and calculated as ETc = ETo × Kc. The ETo (reference evapotranspiration) and tomato crop coefficient (Kc) were determined according to Hargreaves and Samani [73] and Allen et al. [74]. Water supplying (equal to 100% ETc) occurred when 40% of total available water was depleted, according to the evapotranspiration method reported by Doorenbos and Pruitt [75]. Finally, a total irrigation volume equal to 3694 m^3^ ha^−1^ of H_2_O was applied in 2020 through 19 supplies, while 23 irrigations were performed in 2021 throughout crop cycle, amounting to 4500 m^3^ ha^−1^ of H_2_O.

Three commercial consortia of microorganisms were evaluated: *Glomus aggregatum*, *G. intraradices*, *G. mossae*, *G. etunicatum*, *Bacillus amyloliquefaciens*, *B. licheniformis*, *B. subtilis*, *B. laterosporus*, *B. mojavensis*, *Trichoderma harzanium*, *T. koningii* (FID) (Fidelius, Intertec, Bibbiena (AR), Italy); *Glomus* spp., *Bacillus* spp., *Streptomyces* spp., *Pseudomonas* spp., *Arthrobotrys* spp., *Monacrosporium* spp., *Paecilomyces* spp., *Myrothecium* spp., *Trichoderma* spp. (EKO) (EKOprop NX (Green Ravenna srl, Ravenna (RA), Italy); *Glomus coronatum*, *G. caledonium*, *G. mosseae*, *G. viscosum*, *Rhizophagus irregularis*, *B. subtilis*, *Streptomyces* spp., *Pichia pastoris*, *Trichoderma harzanium*, *T. viride* (MIC) (Micosat F Tab Plus (CCS, Quart (AO), Italy) (Supplementary Materials Table S1).

These products, hereafter referred to as FID, MIC and EKO, were compared with untreated control (CTRL). Treatments were carried out at two times: 48 h before transplant and at 10 days after transplanting (DAT). The first application was carried out in plant nursery by dipping the seedlings (raised in cellular containers) up to the collar in an aqueous solution for each of the three commercial blends at these concentrations: 0.2 g L^−1^ for FID or MIC and 0.1 g L^−1^ for EKO. Ten days after transplanting, a second inoculum was performed supplying the commercial microorganism consortia through drip irrigation (without fertilizers dissolved in water) at doses of 1 kg ha^−1^ for FID and EKO and 2 kg ha^−1^ for MIC.

Weed control and plant protection were performed according to the cultivation protocols of the Apulia region (Italy).

A randomized block design with three replications was realized and each plot, containing 20 plants, measured 3.8 m × 1.8 m (6.84 m^2^).

### 4.4. Yield and Merceological Assessment

Manual harvestings were carried out on 2 September in 2020 (106 DAT) and on 6 August in 2021 (99 DAT), when about 90% of fruits were ripe. Total yield (TY) was assessed sorting and weighting the fruits in three commercial categories: MY = marketable yield, GY = green yield (unripe fruits) and rotten yield (RY). The RY to TY (RY/TY), GY to TY (GY/TY) and MY to TY (MY/TY) ratios were also reported in percentage. Mean fruit weight (FW) was evaluated on 100 red-ripe fruits randomly chosen from each plot. The same sample was used to determine the incidence (%) of sunscald (SsF) and fruits infected by TSWV (*Tomato Spotted Wilt Orthotospovirus*) (VrF) showing typical chlorotic blotches and ringspots as symptoms.

### 4.5. Technological Characteristics

Dry matter content (DM) was determined drying 20 g of homogenized tomato sample in a stove at 72 °C until constant weight. The soluble solids content (SSC) was assessed using a digital refractometer (Refracto 30PX, Mettler-Toledo, Novate Milanese, Milan, Italy), and results were expressed as °Brix. The pH and titratable acidity (TtA) were evaluated using the pH-Matic 23^®^ titroprocessor, equipped with a pH electrode (model 5011T) (Crison Instruments, Barcelona, Spain). Titratable acidity was expressed as g of citric acid 100 g^−1^ juice [76,77].

### 4.6. Fruit Metabolic Profiling

Thirty well-ripened fruits per plot were washed and dried, and then sliced and homogenized in a Waring blender (2 L capacity, Model HGB140, PartsTown, Addison, IL, USA) for 1 min, then shock frozen in liquid nitrogen and transported on dry ice to the laboratory of Plant Crop Physiology of University of Campania “Luigi Vanvitelli,” where they were ground to a fine powder in liquid nitrogen and either used immediately for assays or stored at −80 °C.

### 4.7. Starch and Soluble Sugars Analysis

Starch and soluble sugars were extracted according to Dell’Aversana et al., 2021 [78], with some modifications. Fresh tomato fruits (20 mg) were submitted to two subsequent extractions with 250 mL ethanol 80% (*v*:*v*) and a final extraction with 150 mL ethanol 50% (*v*:*v*) at 80 °C for 20 min. The tubes were cooled in ice and centrifuged at 14,000× *g* for 10 min at 4 °C. The clear supernatants of the three following extractions were pooled together and stored at −20 °C until analysis. The pellets of the ethanolic extraction were heated at 90 °C for 2 h in 500 μL of 0.1 M KOH [79]. After cooling on ice, the samples were acidified to pH 4.5, mixed 1:1 with a hydrolysis buffer containing sodium acetate 50 mM pH 4.8, α-amylase 2 U/mL and amyloglucosidase 20 U/mL, and incubated at 37 °C for 18 h. The samples were centrifuged at 14,000× *g* for 10 min at 4 °C, and the supernatant containing the glucose derived from starch hydrolysis was used for measurement. The content of glucose, fructose and sucrose in the ethanolic extracts and the glucose derived by starch were determined by an enzymatic assay coupled with reduction of pyridine nucleotides and the increase in absorbance at 340 nm was recorded using a Synergy HT spectrophotometer (BioTEK Instruments, Bad Friedrichshall, Germany) as described in Carillo et al., 2019 [45]. The content of sugars was expressed as mg g^−1^ DW (dry weight).

### 4.8. Polyphenols and Lycopene Analysis

The total polyphenol content was determined by using the Singleton–Folin–Ciocalteu [80] method with some modifications. An aliquot (50 mg) of tomato samples was suspended in 700 µL of 60% methanol (*v*:*v*), then centrifuged at 25 °C for 10 min at 13,000× *g*. Aliquots of the clear supernatant (35 µL) were added to 125 µL of the Folin–Ciocalteu reagent diluted with H_2_O milli Q (Merck Millipore, Burlington, MA, USA) (1:1 *v*:*v*) and 650 µL of 3% (*w*:*v*) sodium carbonate. After 90 min of reaction at room temperature, the absorbance at 760 nm was determined by a Synergy HT spectrophotometer (BioTEK Instruments, Bad Friedrichshall, Germany). The total polyphenol content in the samples was evaluated with a standard curve obtained using known concentrations of gallic acid (GAE) as a standard. Total phenols were expressed as mg GAE g^−1^ DW. Lycopene concentration (mg g^−1^ DW) was evaluated according to Sadler et al., 1990 [81], with the modifications described in Carillo et al., 2019 [45]. An aliquot of tomato samples (50 mg) was suspended in 380 μL of hexane:acetone:methanol (2:1:1 *v*/*v*), containing 0.05% (*w*/*v*) BHT (butylated hydroxytoluene) to minimize oxidation. Blanks were prepared without tomato extracts. The suspensions were mixed and continuously agitated on an orbital shaker for 30 min. Samples were then centrifuged at 4 °C for 10 min at 14,000× *g*, and an aliquot of 100 μL of the organic phase of orange color was transferred in an Eppendorf tube and 1.4 mL hexane was added. The absorbance at 472 nm was measured by a microplate reader. Lycopene concentration was estimated for comparison with standard curves of pure lycopene and expressed as mg g^−1^ DW.

### 4.9. Soluble Proteins and Free Amino Acid Analysis

Soluble proteins were extracted by mixing 20 mg of fresh tomato fruit with a buffer containing 200 mM TRIS-HCl pH 7.5 and 500 mM MgCl_2_ at 4 °C for 24 h. The clear supernatants (10 µL) were added to 190 µL of Protein Assay Dye Reagent Concentrate (Bio-Rad, Milan, Italy) diluted with H_2_O milli Q (1:5 *v*:*v*). The soluble protein content in the samples was calculated by comparison with standard curves obtained using known concentrations of bovine serum albumin (BSA) as the reference standard [45]. Proteins were expressed as mg g^−1^ DW. Free amino acids were extracted from 40 mg of tomato samples in 1 mL ethanol:water (40:60 *v*:*v*) overnight at 4 °C and estimated by HPLC after pre-column derivatization with *o*-phthaldialdehyde (OPA) according to Dell’Aversana et al., 2021 [78]. Proline was determined in the same ethanolic extract according to Carillo et al., 2019 [45], and expressed as µmol g^−1^ DW.

### 4.10. Data Analysis

All data were submitted to analysis of variance (one-way ANOVA) by using GENSTAT 17th software package (VSN International, Hemel Hempstead, UK). Tukey test was used to separate means, when the F test of ANOVA for treatment was significant at *p* < 0.05. The MY/TY, RY/TY and GY/TY ratios, expressed as percentage, were submitted to Arcsin transformation before ANOVA analysis.

A heat map was generated in Excel, summarizing the responses of the two year cultivation and the three biostimulant treatments. Results were calculated as the logarithm base 1.2 (Log_1.2_) ratio of biostimulant treatments to control plants. Results were visualized using a false color scale with red indicating an increase and blue a decrease, whereas white squares indicated no differences [82].

## 5. Conclusions

Microbial-based biostimulants represent a promising means to improve yield and quality in the processing tomato crop. Several commercial products based on AMF, *Trichoderma* and PGPB consortia are continuously released onto the market, making it necessary to evaluate their effectiveness in different environments and growing conditions. Our findings revealed no significant effect of MIC, EKO and FID treatments on the yield, its components and most technological traits. Despite the significant influence of the year of cultivation, which was mainly related to phytopathological problems affecting the crop in 2020, over the two years the FID biostimulant enhanced several evaluated parameters (protein content, alanine, aspartic acid, γ-aminobutyric acid, glutamine, glycine, monoethanolamine, total amino acids, essential amino acids and branched-chain amino acids) of tomato fruits with respect to the control. Furthermore, all the biostimulant products enhanced the soluble solids content in 2020. Based upon our evidence, FID appears to be the best microbial inoculant to enhance primary metabolites (proteins and amino acids) in the processing tomato fruit (cv “Heinz 1534”) under southern Italian growing conditions.

The results of this study contribute to obtaining more applied insights into the use of microbial-based biostimulants capable of improving the nutritional and functional quality of the processing tomato crop. However, due to the complexity of the applied microbial consortia, it remains to be clarified whether it is useful to apply formulates containing more than three to four microorganisms (including fungi, bacteria and others). In fact, microorganisms live in dynamic equilibrium in the soil and compete for space and resources. This aspect should therefore be investigated and clarified.

## Figures and Tables

**Table 1:** Effects of treatments (MIC = Micosat F Tab Plus, EKO = EKOprop NX, FID = Fidelius, CTRL = control), year and treatment × year interaction on yield components and fruit defects (cv “Heinz 1534”).

	RY	GY	MY	TY	RY/TY	GY/TY	MY/TY	FW	SsF	VrF	TDF
	(t ha^−1^)	(t ha^−1^)	(t ha^−1^)	(t ha^−1^)	(%)	(%)	(%)	(g)	(%)	(%)	(%)
**Treatment**											
MIC	4.61	19.68	75.80	100.10	4.67	19.35	75.98	71.45	3.00	17.17	20.17
EKO	4.70	20.94	79.80	105.43	4.65	19.19	76.16	69.06	4.67	14.00	18.67
FID	5.45	24.18	82.05	111.67	4.90	21.34	73.75	71.17	4.17	15.00	19.17
CTRL	4.97	12.93	83.18	101.08	4.93	12.49	82.58	67.96	5.83	12.50	18.33
Year											
2020	6.13 a	13.55 b	80.89	100.58	6.09 a	13.02 b	80.89 a	68.19 b	5.83 a	26.50 a	32.33 a
2021	3.73 b	25.31 a	79.52	108.56	3.48 b	23.17 a	73.34 b	71.63 a	3.00 b	2.83 b	5.83 b
**Treatment × Year**											
MIC 2020	6.26	10.85	79.42	96.54	6.45	11.15	82.40	69.54	3.00	32.33	35.33
EKO 2020	6.23	19.25	78.14	103.62	6.21	17.31	76.48	67.82	7.00	26.00	33.00
FID 2020	6.29	15.39	82.02	103.70	6.01	14.90	79.09	70.55	5.67	25.00	30.67
CTRL 2020	5.74	8.72	83.98	98.45	5.70	8.70	85.60	64.86	7.67	22.67	30.33
MIC 2021	2.96	28.52	72.18	103.65	2.89	27.56	69.56	73.36	3.00	2.00	5.00
EKO 2021	3.16	22.62	81.46	107.24	3.09	21.07	75.84	70.29	2.33	2.00	4.33
FID 2021	4.60	32.96	82.08	119.64	3.80	27.78	68.42	71.79	2.67	5.00	7.67
CTRL 2021	4.19	17.15	82.37	103.70	4.15	16.29	79.56	71.06	4.00	2.33	6.33
**Significance**											
Treatment	ns	ns	ns	ns	ns	ns	ns	ns	ns	ns	ns
Year	*	**	ns	ns	**	**	*	*	*	***	***
Treatment × Year	ns	ns	ns	ns	ns	ns	ns	ns	ns	ns	ns
**Mean**	4.93	19.43	80.21	104.57	4.79	18.10	77.12	69.91	4.42	14.67	19.08

RY = rotten yield, GY = green yield, MY = marketable yield, TY = total yield, RY/TY = RY to TY ratio, GY/TY = GY to TY ratio, MY/TY = MY to TY ratio, FW = average fruit weight, SsF = sunscald fruits, VrF = fruits with viral symptoms, TDF = total defective fruits. ns, *, **, *** = non-significant or significant at *p* ≤ 0.05, 0.01 and 0.001, respectively. Different letters within each column indicate significant differences between treatments, year and treatment × year according to Tuckey’s range test (*p* ≤ 0.05).

**Table 2:** Effects of treatments (MIC = Micosat F Tab Plus, EKO = EKOprop NX, FID = Fidelius, CTRL = control), year and treatment × year interaction on chemical and technological characteristics of tomato fruits (cv “Heinz 1534”).

	Glc	Fru	Suc	Tsu	Sta	PP	Lyc	pH	TtA	SSC	DM
	(µg g^−1^)	(µg g^−1^)	(µg g^−1^)	(µg g^−1^)	(µg g^−1^)	(µg mg^−1^)	(mg g^−1^)		(g% Citric Acid)	(°Brix)	(g)
**Treatment**											
MIC	12.63	9.06	0.73 a	22.42	6.27	0.29	6.77 ab	4.44	0.48	5.35	6.28
EKO	12.11	9.52	0.27 b	21.90	6.52	0.31	7.99 a	4.52	0.44	5.33	6.33
FID	12.05	9.47	0.64 a	22.15	5.98	0.30	7.04 ab	4.48	0.47	5.38	6.32
CTRL	12.08	9.88	0.48 ab	22.44	6.31	0.30	6.56 b	4.50	0.45	5.17	6.19
**Year**											
2020	11.58	8.20 b	0.82 a	20.60 b	4.69 b	0.29	6.11 b	4.41 b	0.51 a	5.41 a	6.53 a
2021	12.86	10.76 a	0.23 b	23.86 a	7.85 a	0.31	8.07 a	4.56 a	0.41 b	5.21 b	6.03 b
**Treat. × Year**											
MIC 2020	12.24	7.80	1.30 a	21.34	4.60	0.26	5.40	4.39	0.54	5.47 a–c	6.58
EKO 2020	12.07	8.24	0.26 c	20.57	5.06	0.30	7.67	4.42	0.47	5.53 ab	6.74
FID 2020	10.61	7.87	1.03 ab	19.50	4.41	0.30	5.59	4.38	0.52	5.60 a	6.56
CTRL 2020	11.39	8.89	0.71 b	20.99	4.71	0.30	5.80	4.44	0.49	5.03 d	6.24
MIC 2021	13.03	10.33	0.16 c	23.51	7.95	0.31	8.15	4.49	0.42	5.23 a–d	5.98
EKO 2021	12.16	10.80	0.27 c	23.23	7.99	0.33	8.30	4.61	0.40	5.13 cd	5.92
FID 2021	13.50	11.07	0.24 c	24.81	7.55	0.30	8.50	4.58	0.41	5.17 b–d	6.07
CTRL 2021	12.77	10.87	0.24 c	23.88	7.91	0.30	7.32	4.56	0.41	5.30 a–d	6.14
**Significance**											
Treatment	ns	ns	***	ns	ns	ns	*	ns	ns	ns	ns
Year	ns	***	***	*	***	ns	***	**	***	*	***
Treat. × Year	ns	ns	***	ns	ns	ns	ns	ns	ns	*	ns
**Mean**	12.22	9.48	0.53	22.23	6.27	0.30	7.09	4.48	0.46	5.31	6.28

Glc = glucose, Fru = fructose, Suc = sucrose, Tsu = total sugars, Sta = starch, PP = polyphenols, Lyc = lycopene, pH, TtA = titratable acidity, SSC = soluble solids content, DM = fruit dry matter. ns, *, **, *** = non-significant or significant at *p* ≤ 0.05, 0.01 and 0.001, respectively. Different letters within each column indicate significant differences between treatments, year and treatment × year according to Tuckey’s range test (*p* ≤ 0.05).

**Table 3:** Effects of treatments (MIC = Micosat F Tab Plus, EKO = EKOprop NX, FID = Fidelius, CTRL = control), year and treatment × year interaction on proteins (mg g^−1^) and free amino acids (µmol g^−1^) of tomato fruits (cv “Heinz 1534”).

	Prot	Ala	Asn	Asp	GABA	Gln	Glu	Gly	MEA	Orn	Pro	Ser	Tyr	TAA	EAA	BCAAs
	(mg g^−1^)	(µmol g^−1^)	(µmol g^−1^)	(µmol g^−1^)	(µmol g^−1^)	(µmol g^−1^)	(µmol g^−1^)	(µmol g^−1^)	(µmol g^−1^)	(µmol g^−1^)	(µmol g^−1^)	(µmol g^−1^)	(µmol g^−1^)	(µmol g^−1^)	(µmol g^−1^)	(µmol g^−1^)
**Treatment**																
MIC	3.60 b	1.82 b	4.36 b	5.08 b	11.16 b	14.33 b	14.14	0.23 c	0.45 b	0.16	0.80	1.06	0.71 b	58.28 b	2.29 b	0.94 b
EKO	3.69 b	2.52 ab	4.97 ab	4.92 b	11.15 b	14.99 b	13.63	0.29 bc	0.44 b	0.14	0.66	1.26	0.72 b	59.70 b	2.59 b	1.09 b
FID	5.30 a	3.06 a	5.50 ab	7.24 a	18.31 a	22.65 a	16.18	0.41 a	0.55 a	0.15	0.71	1.47	1.02 a	82.71 a	3.42 a	1.48 a
CTRL	3.45 b	2.63 ab	6.20 a	5.52 b	10.37 b	14.60 b	14.78	0.32 b	0.46 ab	0.15	0.73	1.23	0.81 ab	61.93 b	2.58 b	1.02 b
**Year**																
2020	2.14 b	3.15 a	6.66 a	4.65 b	10.22 b	13.71 b	12.87 b	0.34 a	0.39 b	0.18 a	0.98 a	1.51 a	0.78	59.63 a	2.72	1.12
2021	5.88 a	1.86 b	3.86 b	6.73 a	15.28 a	19.57 a	16.49 a	0.28 b	0.56 a	0.12 b	0.47 b	1.00 b	0.86	71.67 b	2.72	1.14
**Treat. × Year**																
MIC 2020	2.30 c	2.10 b–d	4.99	4.16 b	8.90 b	10.89 b	11.05	0.22 b	0.41 bc	0.19 a	1.17 a	1.16	0.64	49.60 b	2.12 b	0.88 b
EKO 2020	2.30 c	3.38 ab	6.50	4.28 b	9.66 b	13.10 b	10.27	0.34 ab	0.34 c	0.15 bc	0.87 b	1.50	0.75	54.90 b	2.51 b	1.07 b
FID 2020	2.13 c	3.23 a–c	7.14	5.18 b	11.98 b	15.73 b	15.01	0.39 a	0.39 bc	0.18 ab	0.90 b	1.72	0.88	67.38 b	3.14 ab	1.28 ab
CTRL 2020	1.85 c	3.92 a	8.00	4.98 b	10.33 b	15.12 b	15.17	0.42 a	0.41 bc	0.19 a	0.99 ab	1.68	0.83	66.67 b	3.11 ab	1.27 ab
MIC 2021	4.90 b	1.54 d	3.72	6.01 b	13.43 b	17.77 b	17.24	0.23 b	0.49 bc	0.12 c	0.43 c	0.95	0.78	66.95 b	2.45 b	1.01 b
EKO 2021	5.09 b	1.67 cd	3.43	5.56 b	12.64 b	16.88 b	16.98	0.23 b	0.53 b	0.13 c	0.45 c	1.03	0.70	64.51 b	2.66 b	1.11 b
FID 2021	8.47 a	2.89 a^–^d	3.86	9.30 a	24.64 a	29.56 a	17.35	0.43 a	0.71 a	0.12 c	0.53 c	1.22	1.16	98.04 a	3.69 a	1.68 a
CTRL 2021	5.05 b	1.34 d	4.41	6.06 b	10.41 b	14.07 b	14.39	0.22 b	0.51 b	0.12 c	0.46 c	0.79	0.80	57.19 b	2.05 b	0.78 b
**Significance**																
Treatment	***	*	*	***	***	***	ns	***	*	ns	ns	ns	**	**	*	*
Year	***	***	***	***	***	***	**	*	***	***	***	***	ns	**	ns	ns
Treat. × Year	***	*	ns	*	***	**	ns	**	**	*	*	ns	ns	**	*	*
**Mean**	4.01	2.51	5.26	5.69	12.75	16.64	14.68	0.31	0.47	0.15	0.72	1.26	0.82	65.65	2.72	1.13

Prot = soluble proteins, Ala = alanine, Asn = asparagine, Asp = aspartic acid, GABA = γ-aminobutyric acid, Gln = glutamine, Glu = glutamic acid, Gly = glycine, MEA = monoethanolamine, Orn = ornithine, Pro = proline, Ser = serine, Tyr = tyrosine, TAA = total amino acids, EAA = essential amino acids, BCAAs = branched-chain amino acids. ns, *, **, *** = non-significant or significant at *p* ≤ 0.05, 0.01 and 0.001, respectively. Different letters within each column indicate significant differences between treatments, year and treatment × year according to Tuckey’s range test (*p* ≤ 0.05).

## Supplementary Materials

The following supporting information can be downloaded at: https://www.mdpi.com/article/10.3390/plants12010014/s1, Table S1: Detailed composition of commercial formulations containing different microbial consortia; Figure S1: The mean maximum and minimum air temperatures and total rainfall during 2020 and 2021 growing seasons.
