# Supplementary material for: Can Microbial Consortium Applications Affect Yield and Quality of Conventionally Managed Processing Tomato?
Source: Plants (Basel). 2022 Dec 20;12(1):14. doi: 10.3390/plants12010014 (PMC9824734; doi:10.3390/plants12010014)
Supplement: Supplementary file 1 [file plants-12-00014-s001.zip › Supplementary Figure.pdf]

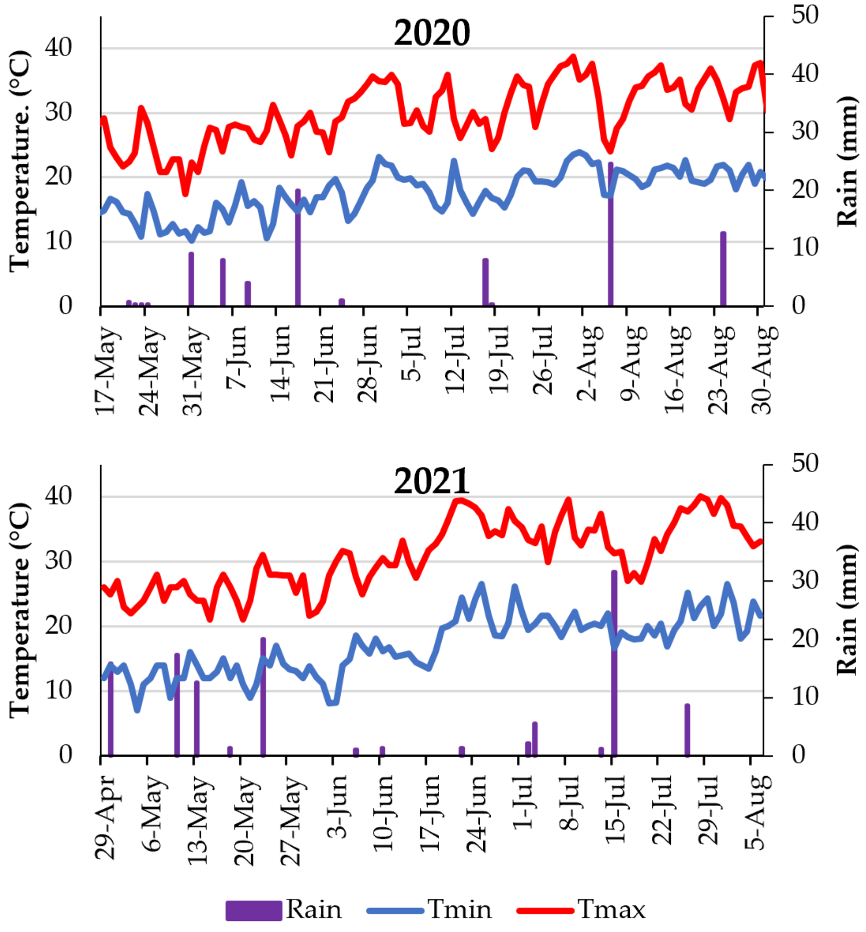

Figure S1. The mean maximum and minimum air temperatures and total rainfall during 2020 and 2021 growing seasons.
