# Supplementary material for: Can Microbial Consortium Applications Affect Yield and Quality of Conventionally Managed Processing Tomato?
Source: Plants (Basel). 2022 Dec 20;12(1):14. doi: 10.3390/plants12010014 (PMC9824734; doi:10.3390/plants12010014)
Supplement: Supplementary file 1 [file plants-12-00014-s001.zip › Supplementary Table.pdf]

Table S1. Detailed composition of commercial formulations containing different microbial consortia.

Commercial Product	Composition	%
**Micosat F Tab Plus (MIC)**	Mycorrhizal fungi (*Glomus coronatum* GU 53, *G. caledonium* GM 24, *G. mosseae* GP 11 e *G. viscosum* GC 41, *Rhizophagus irregularis* RI 31)	10.0%
	Rhizobacteria (*B. subtilis* BA 41, *Streptomyces* spp. SB 19)<br>Yeasts (*Pichia pastoris* PP59)<br>Trichoderma fungi (*Trichoderma harzanium* TH01 and *T. viride TV03*)	7.5%<br>10.2 x 10^7 CFU g^{-1}
**EKOprop NX (EKO)**	Mycorrhizal fungi (*Glomus* spp.)	1%
	Rhizobacteria (*Bacillus* spp., *Streptomyces* spp., *Pseudomonas* spp.)	2.3x10^6 CFU g^{-1}
	Rhizosphere fungi (*Arthrobotrys* spp., *Monacrosporium* spp., *Paecilomyces* spp., *Myrothecium* spp.)	1x10^6 CFU g^{-1}
	Trichoderma fungi (*Trichoderma* spp.)	5x10^6 CFU g^{-1}
**Fidelius (FID)**	Mycorrhizal fungi (*Glomus aggregatum, G. intraradices, G. mossae, G. etunicatum*)	12.0%
	Rhizobacteria (*Bacillus amyloliquefaciens, B. licheniformis, B. subtilis, B. laterosporus, B. mojavensis*)	6x10^6 CFU g^{-1}
	Trichoderma fungi (*Trichoderma harzanium, T. koningii*)	1.5x10^4 CFU g^{-1}
